# Low Complexity Compression and Speed Enhancement for Optical Scanning Holography

**DOI:** 10.1038/srep34724

**Published:** 2016-10-06

**Authors:** P. W. M. Tsang, T.-C. Poon, J.-P. Liu, T. Kim, Y. S. Kim

**Affiliations:** 1 Department of Electronic Engineering, City University of Hong Kong, 83 Tat Chee Avenue, Kowloon, Hong Kong; 2 Bradley Department of Electrical and Computer Engineering, Virginia Tech, Blacksburg, Virginia 24061 USA; 3 Department of Photonics, Feng Chia University, No. 100 Wenhwa Rd., Taichung 407, Taiwan; 4 Department of Optical Engineering, Sejong University, 98 Kunja-dong, Kwangjin-gu, Seoul 143-747 South Korea

## Abstract

In this paper we report a low complexity compression method that is suitable for compact optical scanning holography (OSH) systems with different optical settings. Our proposed method can be divided into 2 major parts. First, an automatic decision maker is applied to select the rows of holographic pixels to be scanned. This process enhances the speed of acquiring a hologram, and also lowers the data rate. Second, each row of down-sampled pixels is converted into a one-bit representation with delta modulation (DM). Existing DM-based hologram compression techniques suffers from the disadvantage that a core parameter, commonly known as the step size, has to be determined in advance. However, the correct value of the step size for compressing each row of hologram is dependent on the dynamic range of the pixels, which could deviate significantly with the object scene, as well as OSH systems with different opical settings. We have overcome this problem by incorporating a dynamic step-size adjustment scheme. The proposed method is applied in the compression of holograms that are acquired with 2 different OSH systems, demonstrating a compression ratio of over two orders of magnitude, while preserving favorable fidelity on the reconstructed images.

Based on a single-pixel optical sensor and a sequential scanning process, optical scanning holography (OSH)^1^,^2^,^3^ is capable of acquiring the complex hologram of a wide-view three-dimensional scene at video rate. From the hologram captured by OSH, the 3-D scene recorded by the hologram can be reconstructed numerically^4^,^5^, or displayed with spatial light modulator. Being different from other techniques in hologram acquisition (such as phase-shifting holography^6^,^7^,^8^), OSH does not require a digital camera to record the hologram. An object scene is scanned in a row-by-row manner, and a hologram pixel is recorded instantaneously at each scan point. An OSH system is intended to be compact, implemented with a simple integration of optical and electronic framework that generally involves only small amount of computing elements. The field of vision of OSH is governed by the coverage of the scanning area, and the acquisition rate determined by the speed of scanning. These imply that the hologram size, and hence the data rate and the time taken to capture a hologram, could be extensive. As such, compression and reducing the number of scan rows are often required to enable efficient transmission of the hologram pixels with a throughput that is compatible with practical constraint.

In the past, it has been demonstrated holographic data can be compressed through popular techniques such as JPEG2000 and JPEG^9^,^10^,^11^ and Vector Quantization (VQ)^12^,^13^,^14^,^15^. On the downside, these methods are computational intensive, and require capturing of the entire hologram prior to compression, hence unsuitable to be incorporated in a compact OSH system. In addition, compression based on VQ requires a codebook that is obtained through a time consuming training process, hence further increasing the complexity of the system. Alternatively, it is also possible to apply the principles of compressive sensing^16^ to acquire a sparse hologram through spiral scanning^17^. Although this could lead to certain degree of reduction in the hologram data, a time consuming iterative optimization process is required to recover the hologram. Straightforward approach based on down-sampling^18^, though simple, is only effective for compressing a hologram by 2 to 4 times. As the compression ratio increases, the degradation to the hologram will become severe. A more promising method is to compress each scanned row of the OSH hologram with Delta Modulation (DM)^19^,^20^,^21^. The method is simple, but suffers the disadvantage of having to estimate a core parameter known as “step size” which is set to certain percentage (e.g., 10%) of the dynamic range of each scanned row of pixels. However, the dynamic range of the hologram pixels is dependent on the object scene, as well as the optical settings (such as depth range of objects and power of the laser beam, etc.) of the OSH system. In refs 19, 20, 21, the step size is determined through trial and error from a set of hologram samples, hence restricting the compression algorithm to a specific OSH system with fixed optical setup. However even if a step size can be estimated through this learning process, it may not be applicable to compressing the entire hologram as each row of the hologram can be very different from the others. Grossly speaking, this technique is not suitable for capturing hologram of arbitrary object scene without prior calibration of the OSH system.

In this paper, we propose a method to overcome the above problems. In the following sections, we shall outline the OSH system and present our proposed method.

## Optical Scanning Holography

**Figure 1:**
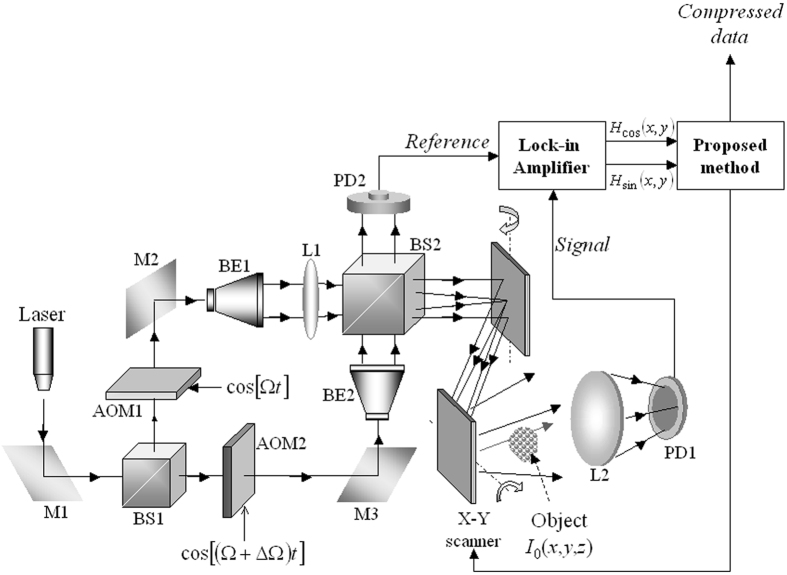
Typical OSH system for acquiring the hologram of an object.

For the sake of completion, a brief outline on the OSH system is presented in this section. Further details on the technology and its recent development can be found in refs 1, 2, 3. We refer to Fig. 1 on a typical OSH system that is used to capture the hologram of a 3-D object with an intensity distribution given by *I*_*o*_ (*x*, *y*; *z*). In the illustrated setup, we have assumed the object is transparent so that the transmitted light is collected by a lens to reach photodetector PD1. If the object is diffusely reflecting, its back-scattered light will then be captured instead. On the right hand side of the figure, we also include the functional block of our proposed method. Suppose the scene is comprising of a 3-D object having an intensity distribution represented by *I*_*o*_ (*x*, *y*; *z*). The object is uniformly scanned by a time-dependent Fresnel zone plate (TD-FZP) beam in a row by row manner with a X-Y scanner. The TD-FZP beam is a combination of 2 optical beams that are generated from a common laser source of frequency *ω*_0_. The 2 beams are upshifted by frequencies Ω, and Ω + ΔΩ with the acousto-optic modulators AOM1 and AOM2, respectively, and collimated by collimators BE1 and BE2. The beam emerges from BE2 is a plane wave at frequency *ω*_0_ + Ω + ΔΩ, while the beam exiting from BE1 is cast on lens L1 to produce a spherical wave at *ω*_0_ + Ω. The TD-FZP, which oscillates at the beat frequency ΔΩ, is formed by combining the plane wave and the spherical wave with beamsplitter BS2 so as to project onto the 3-D object through the X-Y scanner. Lens L2 collects the optical signal scattered by the object and focuses the energy onto photodetector *PD*_1_ to form an electrical information signal at the heterodyne frequency ΔΩ. Photodetector *PD*_2_ gives a heterodyne frequency as a reference signal. Both of the outputs from the photodetectors are delivered to the lock-in amplifier to give the in-phase and the quadrature (Q)-phase outputs, resulting in a cosine hologram *H*_cos_ (*x*, *y*) and a sine hologram *H*_sin_ (*x*, *y*) that together form a complex digital hologram *H*(*x*, *y*) given by


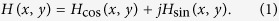


In a typical OSH system, the scanning is conducted through mechanical-driven scanning mirrors. As such the scanning speed is lengthy, and the data rate is high for scanning a wide view scene. Moreover, different OSH systems can have different optical settings (such as power of laser and depth range of object scene) that could affect the dynamic range of the hologram, and hence the compression process that follows. In the following section, we shall address these problems with our proposed compression method.

## Proposed Compression Method

### Scanning and compression of hologram rows

**Figure 2:**
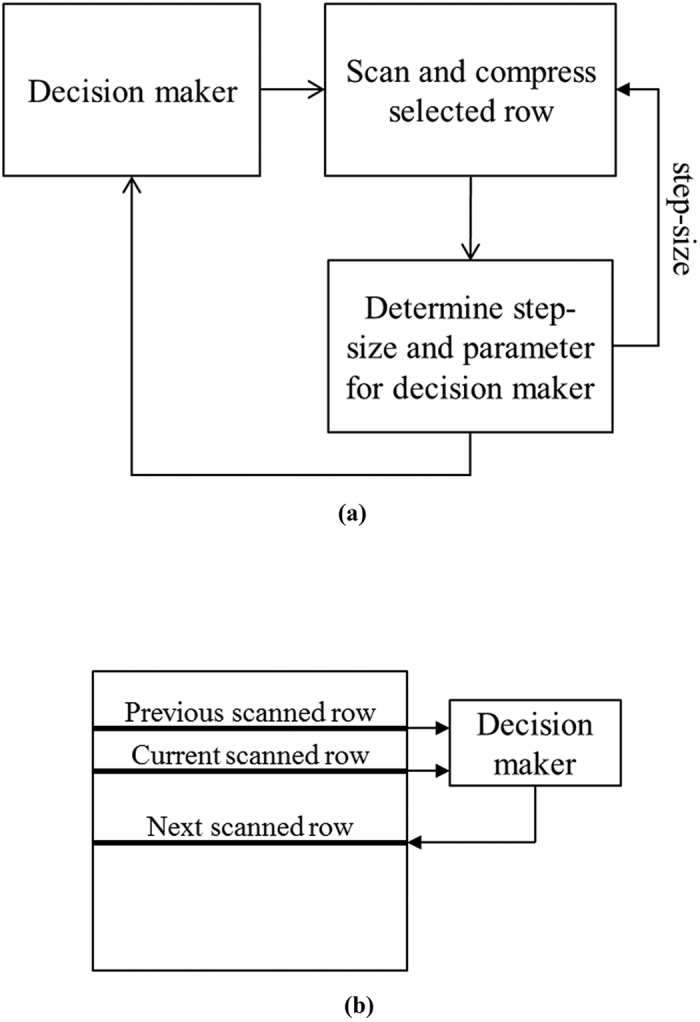
(**a**) Framework of our proposed compression method, (**b**) Example showing predicting the next scan row from the current and previous scanned row by the Decision maker.

Referring to Fig. 1, which shows the classical OSH system and the inclusion of the functional block of our proposed method. An overview framework of our proposed compression method is shown in Fig. 2(a). Briefly, we have employed the concept of adaptive optical scanning holography (AOSH)^22^, whereby a decision maker is employed to select the next hologram row to be scanned, based on the difference between the current and the previous scanned rows as shown in Fig. 2(b). In the course of scanning of each selected row of the hologram, each scanned pixel is compressed with delta modulation, and the parameters required for the decision maker and the step-size for compressing of the next scanned row are determined in an autonomous manner. Being different from the classical OSH system which requires scanning of every rows of the hologram, fewer number of rows are being scanned with our proposed method, hence saving the time taken to acquire a hologram.

We denote each row of the scanned hologram pixels by *H*(*x*, *s*(*τ*)), where 0 ≤ *τ* < *M* is the index of the scanned row of hologram pixels, and *M* is the total number of rows that will be scanned, and is an unknown quantity to start with. The vertical position of the *τ*^*th*^ scanned row is denoted by *s*(*τ*). The horizontal and vertical extents of the hologram and the object scene are assumed to be identical, and given by *X* and *Y*, respectively.

**Figure 3:**
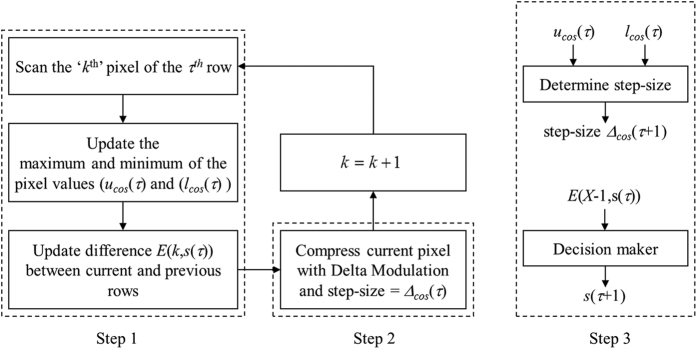
Compression and processing of a row of scanned pixels.

Details of our proposed method are described as follows. The first row *H*(*x*, 0) and the second row *H*(*x*, 1) of hologram pixels are always scanned (i.e., *s*(0) = 0 and *s*(1) = 1). For the first row (i.e., *τ* = 0), the step-sizes *Δ*_cos_ (*τ*) and *Δ*_sin_ (*τ*), which are required to compress the cosine and sine holograms with delta modulation, respectively, are both set to an arbitrary value of 0.1 V, where V is the maximum signal amplitude that can be reached in the system. In the scanning of the each row, there are 2 steps for each scanned pixel to determine the parameters for the decision maker and the DM compression process. After a row of hologram pixels have been scanned and compressed, a 3^th^ step is conducted to deduce the position and step-size of the next scan row. For simplicity of description, we shall only present the process of compressing the cosine hologram, with the understanding that the same will be performed for the sine hologram. The process is shown in Fig. 3. We shall now describe the 3 steps in detail.

Step 1: This step is comprised of 2 operations. First, the maximum and minimum values, i.e., *u*_cos_ (*τ*) and *l*_cos_ (*τ*), respectively, of the pixels in the cosine hologram is updated for each newly scanned pixel as


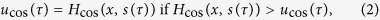



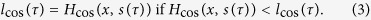


**Figure 4:**
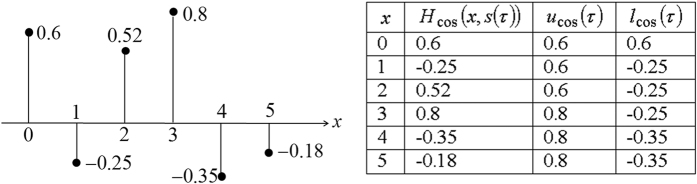
An example showing the updating of the maximum and minimum pixel values in a row of hologram pixels.

Initially, *u*_cos_ (*τ*) and *l*_cos_ (*τ*) are set to V and −V, respectively for *x* < 0, and the updating of the maximum and minimum values is conducted from the first (*x* = 0) to the last (*x* = *X* − 1) scanned pixel. An example of updating *u*_cos_ (*τ*) and *l*_cos_ (*τ*) for the first six pixels of the *τ*^*th*^ row is shown in Fig. 4, assuming that *V* = 1.

Next, the difference between current and previous scanned rows is updated as


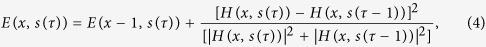


where *E*(*x*, *s*(*τ*)) = 0 for *x* < 0. Note that in Eq. 4, a small memory buffer is required to store a line of the previous scanned row of pixels. In addition, the denominator of the right-hand-side normalized the difference of each pair of pixels to the range [0, 1].

Step 2: One out of every 2 scanned pixel in the cosine holograms is compressed into a 1-bit representation with delta modulation, resulting in a binary sequences *B*_cos_ (*x*, *s*(*τ*)) as given by the following recursive equation:


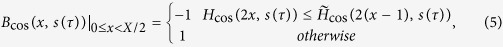



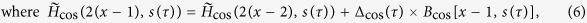


and 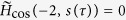. It can be inferred that with Eq. 6 that 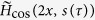 is in fact de-compression of the hologram pixels from the compressed binary sequence *B*_cos_ (*x*, *s*(*τ*)), and only even pixels are included. As such, the number of samples in 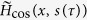 and *B*_cos_ (*x*, *s*(*τ*)) is only half of that of *H*_cos_ (*x*, *s*(*τ*)), equivalent to down-sampling the hologram by 2 times horizontally. An example showing the compression of the six pixels in Fig. 4 is shown in Table 1. Note that only the even pixels at *x* = 0, 2, 4 are compressed while the odd samples are discarded. From Table 1, we can see that only 3 out of the original 6 pixels (the pixel at *x* = −2 is a virtual pixel that is only used in the compression process, but does not exist in the input signal or the compressed data) are being included and compressed due to the down-sampling mechanism. The step-size is assumed to be 0.25.

Step 3: Determine position and the step-size for the next scan row.

Upon scanning and compressing the current row of pixels, the root-mean-square (RMS) value of the mean difference between the current and the previous scanned rows is computed as


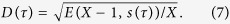


The RMS value *D*(*τ*) reflects the smoothness between a pair of hologram rows. A small value of *D*(*τ*) implies generally smooth variation of the hologram along the vertical direction, so the interval between the current and the next scan rows can be increased, and vice versa. The position of the next scan row is now determined adaptively by the decision maker as


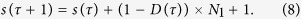


The separation between the current and the next scan row will be within the range from [1, (*N*_1_ + 1)]. The larger the values of *N*_1_, the fewer will be the number of scan rows, but more degradation will be imposed on the hologram. Next, the step size for compressing the next row of the cosine hologram is determines as


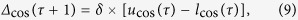


where *δ* < 1. Eq. 9 adjusts the step-size to a fraction *δ* of the dynamic range of the current scan row. The compressed data for each scanned row is comprising of its position *s*(*τ*), the compressed sequences *B*_cos_ [*x*, *s*(*τ*)] and *B*_sin_ [*x*, *s*(*τ*)], and the step-size values Δ_cos_ (*τ*) and Δ_sin_ (*τ*). Disregarding the inclusion of Δ_cos_ (*τ*), Δ_sin_ (*τ*), and *s*(*τ*) that are negligible in data-size as compared with that of the compressed bit-stream, the compression ratio is given by


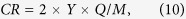


where *Y* and *Q* are the total number of rows, and the number of bits representing each pixel in the original hologram, respectively. *M* is the number of rows that are actually scanned with our proposed method. The factor ‘2’ on the right-hand-side of Eq. 10 is resulted from the compression of only one out of 2 scanned pixels.

### Recovering hologram from compressed data

The de-compression process is conducted in 2 stages. First, the hologram pixels in each scanned row are recovered with Eq. 6, resulting in a row of hologram pixels 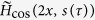. The missing odd pixels are interpolated from a pair of adjacent even pixels as


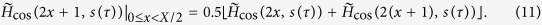


Next, the area in between consecutive scan rows are filled up with bi-linear interpolation as


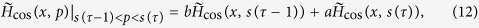


where *a* = [*p* − *s*(*τ* − 1)]/[*s*(*τ*) − *s*(*τ* − 1)] and *b* = (1 − *a*).

## Experimental Results

**Figure 5:**
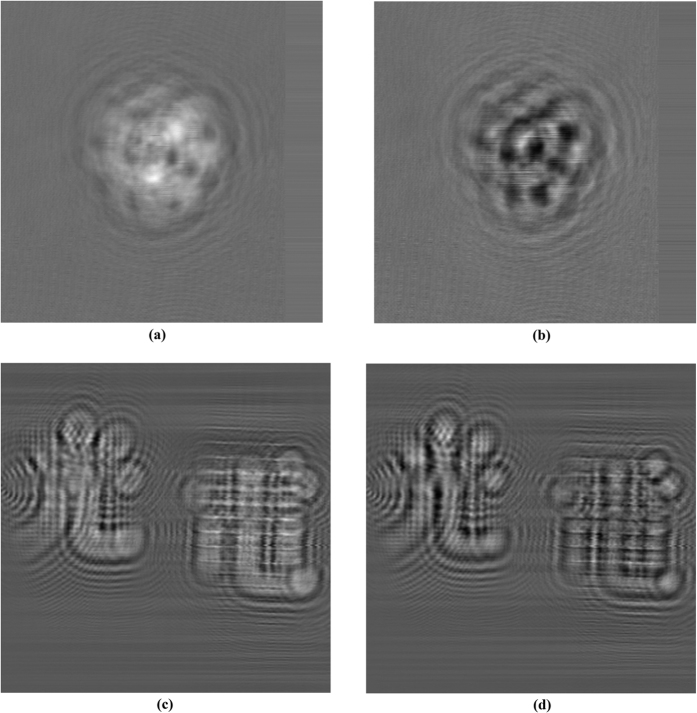
(**a**) Cosine hologram of “A”, (**b**) Sine hologram of “A”, (**c**) Cosine hologram of “B”, (**d**) Sine hologram of “B”.

**Figure 6:**
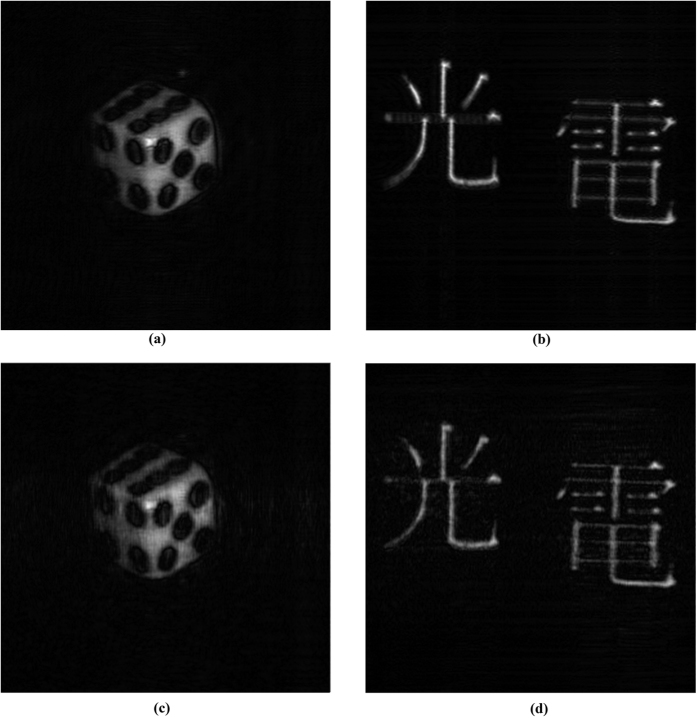
(**a**,**b**) Reconstructed image of original holograms of “A” and “B”, (**c**,**d**) Reconstructed image of compressed holograms of “A” and “B”.

Our proposed method is evaluated with the hologram of 2 objects “A” (a dice) and “B” (2 Chinese characters) that are captured with 2 different OSH systems. The cosine and sine holograms of the objects, acquired with the OSH system based on the optical settings shown in Table 2, are shown in Fig. 5(a–d). The numerical reconstructed images of the 2 holograms at the focused plane are shown in 6(a,b). Next, we applied our proposed method to capture and compress the holograms of the 2 objects with *N*_1_ = 10 and *δ* = 0.1. The numerical reconstructed images of the de-compressed holograms are derived and shown in Fig. 6(c,d). The number of scanned rows, the compression ratios, and the fidelity of the reconstructed images as compared with the original ones shown in Fig. 6(a,b) are listed in Table 3. We have observed that the number of scanned rows have been reduced by about 3.6 times for both objects, resulting in a speed up of the hologram acquisition time by similar factor. For both cases, compression ratio of over 100 times are attained. Apart from slight blurriness, the reconstructed images of the compressed holograms are similar to those of the original holograms, and a high correlation score and peak-signal-to-noise (PSNR) of over 0.92 and 29 dB, respectively, are noted.

## Conclusion

In this paper, we have presented a method for increasing the speed of hologram acquisition in an optical scanning holography system, as well as compressing the holographic data. Our proposed method has 4 major advantages. First, the time taken to scan a hologram is reduced by over 3.5 times. Second, a compression ratio of over 2 orders of magnitude is acheved. Third, our proposed method can be applied to different OSH systems without prior knowledge on their optical settings, rendering the proposed technique being robust. Fourth, the quality of the reconstructed images of the compressed holograms are favorable, exhibiting a high correlation score of over 0.92 as compared with the ones obtained from the original holograms.

## Additional Information

**How to cite this article**: Tsang, P. W. M. *et al*. Low Complexity Compression and Speed Enhancement for Optical Scanning Holography. *Sci. Rep.* **6**, 34724; doi: 10.1038/srep34724 (2016).

## Figures and Tables

**Table 1:** Example of compressing the six hologram pixels in Fig. 4 with delta modulation.

*x*	*H*_cos_ (2*x*, *s*(*τ*))	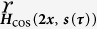	*B*_cos_ (*x*, *s*(*τ*))
−2	—	0	—
0	0.6	0.25	1
1	0.52	0.50	1
2	−0.35	0.25	−1

**Table 2:** Optical setups in the pair of OSH systems.

Object	Depth	Hologram size	Pixel size	Bits per pixel (Q)
A	0.20 m	500 × 500	10.583 um × 10.583 um	16
B	0.022 m	512 × 512	5 um × 5 um	16

**Table 3:** Scanning and compressing test objects “A” and “B” with our proposed method.

Object	Percentage of row scanned, as compared with the original hologram	Compression ratio	Correlation score/PSNR (dB)
A	27.80%	115	0.974/33.46 dB
B	27.54%	116	0.921/29.02 dB
